# Single Molecule FRET: A Powerful Tool to Study Intrinsically Disordered Proteins

**DOI:** 10.3390/biom8040140

**Published:** 2018-11-08

**Authors:** Sharonda J. LeBlanc, Prakash Kulkarni, Keith R. Weninger

**Affiliations:** 1 Department of Physics, North Carolina State University, Raleigh, NC 27695, USA; sleblanc@live.unc.edu; 2 Department of Chemistry, University of North Carolina at Chapel Hill, Chapel Hill, NC 27599, USA; 3 Department of Medical Oncology and Therapeutics Research, City of Hope National Medical Center, Duarte, CA 91010, USA; pkulkarni@coh.org

**Keywords:** single molecule biophysics, FRET, intrinsically disordered protein, IDP

## Abstract

Intrinsically disordered proteins (IDPs) are often modeled using ideas from polymer physics that suggest they smoothly explore all corners of configuration space. Experimental verification of this random, dynamic behavior is difficult as random fluctuations of IDPs cannot be synchronized across an ensemble. Single molecule fluorescence (or Förster) resonance energy transfer (smFRET) is one of the few approaches that are sensitive to transient populations of sub-states within molecular ensembles. In some implementations, smFRET has sufficient time resolution to resolve transitions in IDP behaviors. Here we present experimental issues to consider when applying smFRET to study IDP configuration. We illustrate the power of applying smFRET to IDPs by discussing two cases in the literature of protein systems for which smFRET has successfully reported phosphorylation-induced modification (but not elimination) of the disordered properties that have been connected to impacts on the related biological function. The examples we discuss, PAGE4 and a disordered segment of the GluN2B subunit of the NMDA receptor, illustrate the great potential of smFRET to inform how IDP function can be regulated by controlling the detailed ensemble of disordered states within biological networks.

## 1. Introduction

**Figure 1:**
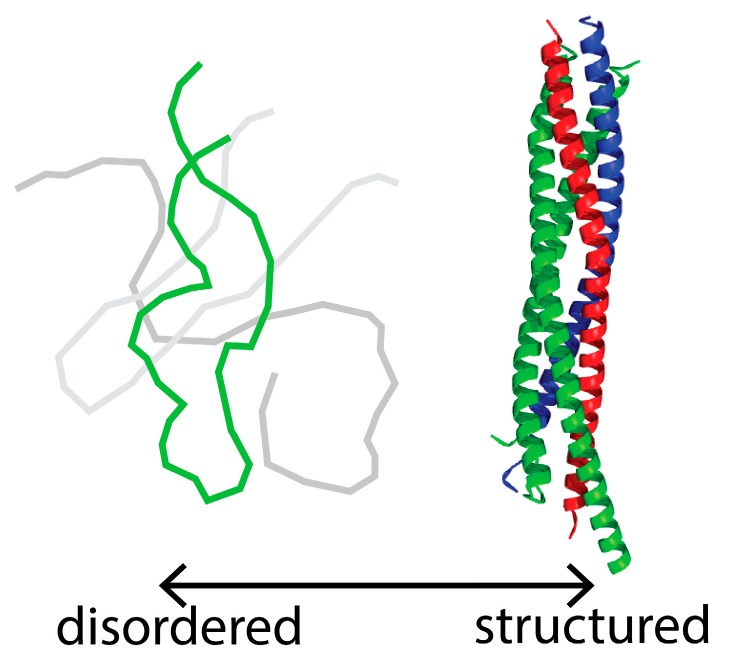
Intrinsically disordered protein (IDP) vs. structured protein. (LEFT) A diagram representing free SNAP-25 (synaptosome-associated protein of 25 kDa, green), which is disordered in isolation. The gray lines represent potential alternative conformations of the protein, consistent with a flexible structure. SNAP-25 folds into a stable structure upon binding its partner SNARE (soluble *N*-ethyl maleimide sensitive factor attachment receptor) proteins. (RIGHT) The SNARE complex [protein databank (PDB) ID: 1SFC)] with synaptobrevin-II (blue), syntaxin-1A (red), and SNAP-25 (green). Note SNAP-25 becomes stably structured within the SNARE complex.

Intrinsically disordered proteins (IDPs) and proteins containing intrinsically disordered regions (IDRs) are increasingly recognized as critical components of cell signaling pathways and regulatory networks [1,2,3,4,5,6]. Unlike the precisely folded, static structures commonly associated with the colorful images of enzymes reproduced in textbooks and on journal covers, IDPs do not fold into a stable structure. Rather, the peptide chain of an IDP continuously fluctuates through a large conformational space (Figure 1). This conformational flexibility, inspiring IDP configuration spaces to sometimes be called pleomorphic ensembles or ‘fuzzy’ structures, is key to the ability of IDPs to serve as hubs in signaling networks. The broad range of interchanging conformations allows IDPs to interact with multiple partners to coordinate a variety of possible signaling pathways. A central role for IDPs in such cellular transactions is reflected in the recent findings that cellular networks have developed switches (i.e., phosphorylation) to control the range of IDP configurations in order to regulate signaling pathways [7,8,9,10]. Thus, elucidating IDP conformational dynamics is likely to uncover the mechanisms by which IDP conformational dynamics can modulate cell signaling.

The rapid and unpredictable conformational dynamics of IDPs presents challenges to experimental approaches to characterize their structures. The average size of IDPs reflected by the drag when moving in a fluid (hydrodynamic radius) can be measured by several methods like analytical ultracentrifugation (AUC), dynamic light scattering (DLS), and gel filtration. Hydrodynamic radius reflects the mobility of the particle in solution, allowing modeling to estimate the spatial extent, and in some cases, information on simple shape parameters (i.e., round vs. oblong). Borrowing ideas from polymer physics, the hydrodynamic radius can be related to physical determinations of the state space of IDPs such as root mean squared end-to-end distance or radius of gyration of the chain, although it is important to emphasize that relations to convert between hydrodynamic radius and radius of gyration are highly non-trivial in most cases. Other, biophysical techniques such as static light scattering, nuclear magnetic resonance (NMR) and small angle X-ray or neutron scattering (SAXS and SANS respectively) can provide access to these parameters and allow experimental comparisons to effective hydrodynamic radius. However, it is important to note that these methods provide information only about the average of an ensemble of configurations and do not provide insights into molecular heterogeneity.

Nonetheless, NMR experiments can provide finer detail about contacts in the chain and ensembles of fluctuations, but are time consuming and tedious [11,12,13]. In contrast, fluorescence-based methods such as fluorescence correlation spectroscopy (FCS), nanosecond FCS (nsFCS), nsFCS-FRET and contact quenching methods provide access to fast timescales (near fluorescence lifetimes that can be in the nanosecond range) and can give important information on local fluctuations, nonlocal contacts, and chain reconfiguration kinetics [14,15,16,17,18,19]. This minireview will focus on single molecule fluorescence (or Förster) resonance energy transfer (FRET) (or smFRET) [20,21] and highlight how this technique can provide real time information about dynamic changes in an IDP ensemble with sensitivity to individual fluctuations or sub-populations. 

Single molecule FRET has several unique advantages over other methods typically used to study IDPs. smFRET has temporal resolution ranging from 0.1 ms to over 1000 s. The ability to follow a single molecule for extended times provides a tool to reveal ensemble switching on timescales that are long compared to molecular fluctuations. The length scales probed by smFRET are in the range of 2–8 nm, which are longer length scales than contact quenching methods, revealing conformations where points on the protein chain being probed for quenching may never come into close contact. Finally, compared to other methods like SAXS or NMR that require extensive equipment, smFRET is relatively inexpensive and easy to implement. Therefore, smFRET provides a quick screening tool to characterize the effects of post-translational modification, sequence variation, or the impact of environmental changes on the global conformational ensemble of IDPs. 

Single molecule FRET is gaining popularity for the study of IDPs, and several excellent reviews have provided insightful perspectives [21,22,23]. In this minireview, we present a practical viewpoint and focus on considerations for designing, executing and interpreting experiments measuring smFRET from IDPs. We conclude by highlighting selected studies that illustrate the use of smFRET to discern IDP structure/function connections.

## 2. How to Apply Single Molecule FRET to Intrinsically Disordered Protein Studies

**Figure 2:**
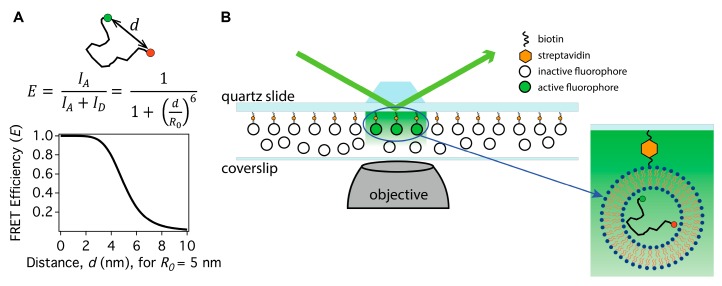
(**A**) The fluorescence (or Förster) resonance energy transfer (FRET) efficiency (*E*) is calculated by the relationship between donor (*D*, green circle) and acceptor (*A*, red circle) intensities (*I_D_* and *I_A_*). FRET efficiency has a strongly non-linear dependence (sixth power) on the distance between the donor and acceptor molecules (*d*) attached to an intrinsically disordered protein. *R*_0_ is the Förster radius, which determines the length scale of the FRET coupling and is the value where the transfer efficiency is 50%. The donor and acceptor fluorescent dye properties determine *R*_0_, which usually is around 4–7 nm. (**B**) A schematic of prism-type total internal reflection (TIR) illumination for smFRET. The green arrow shows an incident laser beam that is totally internally reflected at the quartz-water interface of a fluidic channel, producing an evanescent wave that excites fluorophores near the surface. The zoomed detail shows an IDP labeled with a D–A pair that is encapsulated in a liposome (not to scale). The liposome is attached to the quartz surface via a biotin-streptavidin linkage while additional liposomes are shown in solution in the flowcell diagram.

FRET is the distance-dependent coupling between two fluorophores with different spectral absorption and emission ranges. One of the fluorophores, typically called the donor, absorbs excitation light. Excited donors that are isolated will emit their characteristic spectrum through usual fluorescence processes. If the donor is sufficiently close to the other fluorophore, called the acceptor, rather than the excited donor emitting its own spectrum, the excitation of the donor can transfer to the acceptor, resulting in acceptor emission. The ratio of the acceptor and donor emission intensities provides information on the separation of the fluorophores (Figure 2A). The specific choice of fluorophores impacts the length scale for which FRET is useful. Thus, initial considerations in any FRET experiment are which fluorescent probes to use and how to attach them to the molecule under study.

Fluorophores for single molecule FRET studies are required to be bright (high quantum yield) and also have long emission lifetimes before photobleaching. Suitable fluorophores are extensively reviewed elsewhere [24], but commonly include the commercially available cyanine (Cy3, Cy5), Alexa and Atto series. These fluorophores are available with several chemical modifications that enable attachment to IDPs. Because cysteine is not common in most proteins, cysteine reactive fluorophores are often used to attach the fluorophores to specific cysteine residues either naturally occurring or introduced by site-specific mutation into a cysteine-free IDP mutant. As with any fluorescence modification, it is important to verify that labeled and unlabeled proteins behave the same in all available functional assays (e.g., ligand binding, ATPase activity, etc.). Whether or not functional assays are available to compare labeled and unlabeled samples, additional experiments to assess possible impacts of labeling are advisable. For example, comparing smFRET measurements for given label attachment sites using different fluorophore pairs can be used to investigate how much influence specific properties of the dyes (dimension, charge, hydrophobicity, etc.) might have on the configurations of the protein being studied. In addition, consistency among experiments using a variety of label attachment locations for any given pair of fluorophores builds confidence that labeling does not fundamentally alter molecular function. An IDP containing two cysteines exposed to a mixture of reactive donor and acceptor fluorophores will result in random labeling, which is typically not a problem for smFRET applications to IDPs. By measuring single molecule level fluorescence emissions, the populations of protein labeled with two donors or two acceptors can be ignored and only the mixed donor/acceptor population carried forward in analysis. Possible environmental-specific effects on the fluorophore behavior due to the specific context of each cysteine are usually negligible for most applications that look for conformational switching or that do not rely on absolute calibration of the distance between the probes. If needed, site-specific labeling of each fluorophore is possible using several approaches including unnatural amino acid incorporation or enzymatic labeling of short target amino acid sequences [25]. Labeling with more than two fluorophores can permit simultaneous measurements of more than one distance along the peptide chain, although these are advanced methods [26,27,28,29,30,31].

Instruments to measure smFRET signals from dye-labeled IDPs excite fluorescence with laser sources and collect emission with high numerical aperture microscope objectives. Background fluorescence must be minimized to allow detection of the dim single molecule signals. Two different optical illumination configurations are commonly used to minimize background fluorescence, total internal reflection (TIR) illumination and confocal microscopy [22,23,32,33]. TIR is applied with IDPs immobilized on the surface of a flow cell. Commonly, molecules that are modified with biotin, 6-histidine tags or other affinity tags are tethered directly to their binding partners that are adhered to a passivated surface (avidin/streptavidin for biotin, antibodies for 6-histidine tags, etc.). IDPs that lack interaction with lipid bilayers can be encapsulated inside lipid vesicles that are tethered to the surface (Figure 2B) [34]. For either surface tethering approach, immobilization has the advantage of allowing observation of a single protein for a long time period, but has the drawback of potentially disruptive surface interactions. Surface interactions are avoided by focusing the beam of a confocal microscope into the bulk of a solution, but molecules are only detected for a time in the millisecond range as they freely diffuse through the laser focus. Strategies based on correlating repeated visits to the imaging volume can extend the useful information extracted in the confocal experiments [35]. In both illumination schemes, fluorescence emission is collected by a microscope objective lens, spectrally divided by a dichroic optic, filtered to isolate the donor and acceptor emission wavelength ranges, and relayed to detectors. The signals from single molecules are low and require detection with the most sensitive devices including electron multiplied CCD (emCCD) and scientific CMOS (sCMOS; or scientific complementary metal-oxide-semiconductor) cameras, avalanche photodiodes (APD) or photomultiplier tubes (PMT). If the illumination is a pulsed light source, fluorescence lifetime measurements may be acquired and converted into FRET efficiencies that relate to donor–acceptor separations. With continuous illumination, the detectors provide intensity as a function of time for the donor and acceptor channels (*I_D_* and *I_A_* respectively), from which the FRET efficiency *E* can be calculated as *E* = *I_A_*/(*I_A_* + *I_D_*) which is related to the instantaneous donor–acceptor separation *d* as *E* = 1/(1 + (*d*/*R*_0_)^6^) (Figure 2A). The parameter *R*_0_, called the Förster radius, determines the length scale of the FRET coupling and is the value where the transfer efficiency is 50%. *R*_0_ is determined by properties of the donor and acceptor fluorescent dyes and is most often in the range of 4–7 nm. This range of Förster radii makes FRET useful for donor–acceptor separations between 3 nm and 8 nm. In some cases, FRET efficiency can be corrected for instrumental and environmental factors to provide quantitative distance information between the donor and acceptor fluorophores and thus can be a valuable tool for structural studies of biological molecules [36,37,38,39].

Intrinsically disordered proteins by definition do not exist in a single, well-defined conformation that would give rise to a single separation distance for attached donor and acceptor dyes. Rather, they exist as ensembles of rapidly interconverting conformers. These dynamic configurational changes are rapid compared to the experimental measurement interval, which results in the smFRET efficiency being averaged over a range of distances [40]. Although this rapid conformational exchange strengthens the highly averaged interpretation of the relative donor-acceptor dipole orientation factor in FRET (the kappa-squared factor, discussed in more detail elsewhere [41,42]), the nonlinearity in the fundamental relation between FRET efficiency and dye separation can lead to substantial uncertainties when evaluating this average. A model of the probability distribution of the distances between the donor and acceptor *P*(*r*) is required to relate the measured averaged FRET efficiency to the underlying donor–acceptor separation, but the appropriate model is not always clear. An averaged FRET efficiency <*E*> can be calculated given a probability distribution (*P*(*r*)) and the FRET efficiency vs. distance relation, *E*(*r*) = 1/(1 + (*r*/*R*_0_)^6^) as

$$\;\langle E\rangle =\int^{}P\left(r\right)E\left(r\right)dr\;$$

The Gaussian distribution for *P*(*r*) is commonly used, but other probability distributions are sometimes included to account for solvent quality, self-avoidance, internal friction or other polymer physics phenomena [40,43,44,45,46,47].

Not strictly a natively disordered state, unfolding of proteins by strong denaturants generates an extended coil conformation that has been studied extensively by smFRET [14,15,20,41,48]. Denaturants unfold proteins by reducing chain-chain interactions and making chain–solvent interactions more favorable, simplifying the energy landscape of the denatured state compared to that governing the conformational ensembles of natively unfolded proteins. Because of the simpler energy landscape in denaturing conditions, the protein configuration is expected to follow predictions of simple homopolymer theories more closely than the native state, with possible corrections for excluded volume, internal friction or global corrections for solvent quality. Most denaturation studies of protein unfolding use the diffusing protein confocal experimental approach because the surface immobilization schemes often rely on affinity from folded proteins like antibodies or streptavidin as well as stable lipid vesicles.

## 3. Do Single Molecule FRET and Small Angle X-ray Scattering Agree about the Size of Intrinsically Disordered Proteins?

Measurements of molecular size for proteins in high concentrations of denaturant using smFRET and scattering methods like SAXS or SANS agree well, but smFRET usually reports compaction of denatured proteins upon removal of denaturant that is often not seen by SAXS/SANS approaches [49,50,51,52]. One notable experiment attempting to address this issue applied both smFRET and SANS to a dual labeled polyethylene glycol (PEG) molecule [53]. The PEG study found the same deviation of smFRET and SANS signals seen in most proteins. The authors characterized this study as a negative control because they suggested that compaction of PEG upon denaturant removal is not expected, although it is established that urea and guanidinium chloride both have favorable interactions with PEG [54] and may affect PEG configuration in aqueous solution [55]. Thus, the PEG study is inconclusive as a negative control if the PEG configuration is altered by denaturant. Several studies of proteins in denaturant have ruled out denaturant effects on the index of refraction of the solvent, effects on dye quantum yield, viscous effects on dye rotation, or spectral shift of dye labels [53,56]. Small angle X-ray or neutron scattering studies with labeled and unlabeled proteins have ruled out compaction due to the presence of the dye modification for select test-protein cases [57]. The source of the discrepancy at low denaturant concentration or native conditions for intrinsic disorder remains controversial.

A resolution of the discrepancy has been suggested to derive from the fact that both methods require substantial modeling to relate the measured data to an estimate of a characteristic polymer parameter like radius of gyration [41,48,57,58]. smFRET requires assumption of a model of the ensemble of molecular conformations (*P*(*r*)) whereas SAXS/SANS require models of scattering. Specifically, a number of groups have reached a variety of conclusions about the source of the discrepancy between the methods applied to denatured proteins or IDPs including underlying heterogeneity in the ensembles beyond random polymers [57], the insufficiency of these models [59,60,61,62], or subtle decoupling of the precise quantities averaged in the two methods of measurement [41,48,57,58]. Yet, this topic remains a point of spirited debate with increasingly sophisticated modeling and data analysis approaches reducing the observed discrepancies [63,64,65].

Despite the systematic difference determined for native state IDP sizes when these more sophisticated corrections are not used, both SAXS/SANS and smFRET can be very effective as tools to report relative shifts in the size of the native state due to biologically relevant changes like exposure to divalent salts or post-translational modifications of proteins. For the IDP PAGE4, which plays an important role in prostate cancer, the radius of gyration (*R*_gyr_) of the nonphosphorylated protein was determined to be 36.2 Å by SAXS and 34 Å by smFRET [66]. Upon phosphorylation by the kinase CLK2, the *R*_gyr_ increased to 49.8 Å by SAXS and to 43 Å by smFRET [66]. Although smFRET produced a systematically smaller *R*_gyr_ estimate, the trends in the change upon phosphorylation are the same and can be useful to qualitatively assess the impact on the IDP ensemble of post-translational modification. The ability of both SAXS and smFRET to detect this change in PAGE4 illustrates one of the most powerful applications of smFRET, as a quick reporter of changes in IDP behavior when studying their function in signaling networks.

## 4. Unique Phenomena Identified by Applying Single Molecule FRET to Intrinsically Disordered Proteins

Single molecule FRET has been applied to many different IDPs and denatured proteins to measure different properties including the size of the expanded state, sensitivity to solution properties, peptide sequence dependence, and suitability of polymer theories such as internal friction of the Rouse chain model or timescales of chain dynamics. Extensive descriptions of these previous applications of smFRET have been provided in several excellent reviews that have been recently published, and we refer the reader to those sources for more details [19,21]. In this minireview, we focus our discussion on the application of smFRET to two biologically relevant phenomena observed in IDPs: spontaneous ensemble switching in disordered systems, and modulation of disordered ensembles by phosphorylation with functional impacts on signaling.

### 4.1. Spontaneous Switching among Intrinsically Disordered Protein Ensembles

As mentioned above, IDPs exist as dynamic ensembles of rapidly interconverting conformers. In the highly denatured state of chemically unfolded proteins, the dominance of the solution interaction over intrachain interactions leads to a relatively barrier-less energy landscape. Such denatured proteins may smoothly explore all parts of the energy landscape, approaching ideal polymer type behaviors. Some natively disordered proteins also reflect this behavior. For example, fluorescence correlation spectroscopy (FCS) analysis of a natively unfolded yeast prion monomer displayed rapid fluctuations in the 20–300 ns timescale, demonstrating rapid transitions within an ensemble of configurations [67]. In many natively disordered proteins, intrachain interactions can be sufficiently strong such that, although they do not lead to a stable fold, they restrict the realized conformations. These interactions can lead to variable degrees of preformed secondary structures. These preexisting favorable conformations are suggested to influence interactions with binding partners and contribute to the spectrum of binding phenomena ranging from induced folding to conformational selection [6]. smFRET has revealed folding/unfolding transitions in the IDP α-synuclein in response to ligand (lipid-mimic) binding or addition of osmolytes to the solution [68]. This result suggests that the energy landscape of IDPs can be subtly balanced between folding and unfolding, with small perturbations to the solution inducing transitions.

Barriers to transitions between different folded and unfolded states are low enough in some systems that they spontaneously switch. smFRET revealed spontaneous dynamics in the disordered regions of ankyrin repeats that likely are folding/unfolding transitions [69,70]. These switching events were modulated by temperature or sequence mutations, suggesting that the energy barriers between folded and disordered states are modest.

The energy landscape of some IDPs appear to have generally flat regions that are isolated from each other by barriers that are sufficiently close to thermal energies to allow jumps between the isolated regions [71]. Thus, these proteins fluctuate within a basin for extended times and occasionally, spontaneously switch among the distinct disordered ensembles in different basins. The rate of switching among these different conformational basins may impact their biological functions by regulating interactions with downstream factors. smFRET is a unique experimental approach that can detect transitions of the disordered state that involve global changes in the extent of disordered states. These states can persist for seconds before switching to a distinct disordered state with a different average size. In smFRET measurements of proteins freely diffusing in solution, use of confocal microscopy ensures proteins spend only brief moments in the sensitive volume so that detection of distinct populations suggests long lived states. Correlation spectroscopy can provide information on the switching timescales, as was demonstrated for the sic1 protein that displays switching among distinct, disordered configurations [72]. smFRET using TIR illumination with immobilized proteins permits observations of the same molecule until photobleaching of the dyes occurs [34] which has been particularly useful to observe long lived states. Cytoplasmic regions of disordered domains of several neuronal proteins including neuroligin and C-terminal domain of *N*-methyl-d-aspartic Acid (NMDA) receptor subunit GluN2B (abbreviated C-term-N2B from here on) were observed to give smFRET signals that indicated spontaneous switching among well separated FRET states that persisted for lifetimes of a few seconds, while other measures of the proteins indicated no formation of folded structures [71]. Similar behavior has been detected by smFRET for the disordered protein 4.1-G, which is an adaptor protein linking the cytoskeleton to membrane proteins [73].

These examples of disordered proteins that do not attain a stable structure, but at the same time have restricted ranges of fluctuations where the distinct ensembles can switch, support conclusions that IDP conformations are not smoothly sampling the entire available state space in the free energy landscape. Rather, substantial barriers within the free energy landscape may transiently restrict exploration of isolated regions of confirmation space. These persistent sub-ensembles likely have differential abilities to interact with binding partners. In this case, controlling the relative populations of the separated conformational spaces could be effective for regulating IDP function in cell networks. Single molecule FRET has been a useful tool for characterizing effects that modulate selection of these sub-ensembles with functional impacts, as we shall discuss in the next section.

### 4.2. Phosphorylation Modulates Intrinsically Disordered Protein Configuration and Function

Post translational modification of IDPs is a major regulatory mechanism that is critical both in the healthy state and in disease [74]. NMR studies and molecular dynamics simulations have characterized how phosphorylation of IDPs can change both coil size (*R*_gyr_) and affinity for binding partners. The RNA-binding protein fused in sarcoma (FUS) provides an illustrative example where NMR was used to correlate phosphorylation-induced impacts on IDP properties with changes in functionality [75]. In that study, IDP behavior persisted in FUS after phosphorylation, but transient domain collapse and self-interaction were reduced. Phosphomimetic versions of FUS, which also were less prone to aggregation than unphosphorylated wild-type FUS, reduced toxicity in live cell models. Thus, while in that study FUS toxicity is associated with aggregation, more generally, phosphorylation of IDPs can control other IDP behaviors without the presence of aggregation. Here we will review two systems for which smFRET has provided insight into regulation of IDP function by phosphorylation with mechanisms that involve changing IDP configurational ensembles.

Single molecule FRET can be used to characterize changes in IDP size upon phosphorylation, and in some cases, these changes have been linked to molecular function of the targeted protein. The C-terminal domain of the GluN2B subunit of the NMDA receptor (C-term-N2B), as mentioned earlier, has been determined by smFRET to spontaneously switch between distinct disordered subensembles [71]. Phosphorylation of this domain by Src kinase is an important mechanism to regulate gating of this ion channel [76]. smFRET studies of C-term-N2B [77] determined that phosphorylation does not eliminate the disordered state, but rather leads to expansion of the disordered configuration of C-term-N2B with spontaneous transitions among sub-ensembles persisting. Surface tethering C-term-N2B in the orientation relevant to its context in the full channel further increased the swelling upon phosphorylation [77]. Because phosphorylation regulates the function of the channel, it was suggested that IDP properties were responsible for this regulatory function.

In a follow up study with C-term-N2B [78], the authors used smFRET measurements to further connect modulation of the disordered state properties to regulation of the channel. The C-terminal IDP domain mediates inhibition of the full NMDA receptor channel by extracellular zinc. Src kinase phosphorylation of the regulatory domain both expands the IDP and eliminates the sensitivity to zinc. The authors used proline depletion near the phosphorylation site to affect the IDP properties. smFRET demonstrated that proline depletion led to compaction of the IDP (in contrast to phosphorylation of the wild-type), but disorder was maintained and the molecules continued to demonstrate slow switching among sub-ensembles although with fewer molecules switching. Zinc inhibition was lost in the proline-depleted version although other channel properties were unaffected. Contrasting the increase in *R*_gyr_ due to phosphorylated WT C-term-N2B to the decrease in *R*_gyr_ of the proline depleted version, given that both produce the same loss of zinc inhibition, suggests a more complex relationship between disordered properties and allosteric regulation of the channel. Perhaps a clue to the mechanism of this connection was revealed when the authors demonstrated that the kinetics of binding of the target ligand PSD-95 to C-term-N2B for the Src phosphorylated WT version (which is different from the nonphosphorylated WT), was unchanged in both the phosphorylated and nonphosphorylated proline-depleted version [78]. This finding points to the importance of the detailed kinetics of transitions within the disordered state for the allosteric mechanisms that impact gating the channel.

Studies of Prostate-associated Gene 4 (PAGE4) are another example where smFRET measurements have contributed to connecting phosphorylation-induced modulation of IDP properties to regulation of downstream cellular signaling. PAGE4 is an IDP [79,80] that is normally only expressed in the testis and in the fetal prostate, but is aberrantly expressed in prostate cancer (PCa). Interactions between PAGE4 and transcription factors AP-1 (c-Jun/c-Fos dimeric transcription factor complex) have been suggested to control cellular phenotypes of PCa cells related to androgen sensitivity [66,81,82,83].

A functional correlation between the phosphorylation-dependent regulation of PAGE4 conformational dynamics and potentiation of transactivation by c-Jun was observed using smFRET to characterize the global size (*R*_gyr_) of PAGE4. Shifts to larger PAGE4 *R*_gyr_ were induced by binding c-Jun while maintaining a disordered state of PAGE4 [80]. Single molecule FRET and NMR determined that phosphorylation of PAGE4 by homeodomain-interacting protein kinase 1 (HIPK1) yields a more compact ensemble of configurations, which restricts the c-Jun binding site and effectively disrupts the PAGE4 interaction with c-Jun [84,85]. Interestingly, HIPK1 phosphorylation stimulates c-Jun dependent transcription. Taken together, these observations suggest that increased transcriptional activity is achieved by disrupting the c-Jun/PAGE4 interaction. These observations suggest that nonphosphorylated PAGE4 binding to c-Jun is sequestering c-Jun from other interactions required for it to stimulate transcription.

A more complete picture of this signaling cascade was revealed with studies of PAGE4 treated with a different kinase, CDC-Like Kinase 2 (CLK2) that leads to hyper-phosphorylation. In contrast to the stimulation of c-Jun activity following PAGE4 phosphorylation by HIPK1, CLK2 phosphorylation of PAGE4 inhibits the stimulation of c-Jun dependent transcription. Combined results from NMR, PRE, SAXS, and smFRET allowed modeling of the ensembles of configurations in the disordered state. This modeling revealed changes in the ensembles due to distinct phosphorylation patterns [66,85]. Full molecular dynamics simulations further elucidated details of the shift in these disordered ensembles for PAGE4 with distinct phosphorylation patterns [86]. HIPK1-PAGE4 exhibits a relatively compact conformational ensemble that binds AP-1 (the full c-Jun/c-Fos dimer), whereas CLK2-PAGE4 is more expanded and resembles a random coil with diminished affinity AP-1.

These kinase-induced alterations in the PAGE4 ensemble, which impact AP-1 potentiation, also regulate androgen sensitivity phenotypes of PCa cells. HIPK1 is expressed in both androgen-dependent and androgen-independent PCa cells, whereas CLK2 and PAGE4 are expressed only in androgen-dependent cells. A model of a PAGE4/Jun-Fos (AP-1)/AR regulatory circuit in PCa cells suggests that the circuit can display oscillations, hinting that androgen dependence may not be a fixed state but can vary temporally [66]. Thus, phenotypic switching between androgen-dependent and androgen-independent states driven by differential phosphorylation of PAGE4 might be an eventual target to guide development of new treatment strategies [81,82,83,86].

These two examples of C-term-N2B and PAGE4 illustrate the potential of smFRET experiments to measure quantitative changes in global structural or kinetic properties of the disordered state of IDPs that can be directly linked to physiological outcomes.

## 5. Future Directions

Our understanding of the ‘fuzzy logic’ with which IDPs regulate biological functions is rapidly increasing. One of the most intriguing discoveries is the ability of IDPs to condense into phase-separated domains within cells and serve as transient organelles lacking structural boundaries (also referred to as proteinaceous membrane-less organelles or PMLOs), like the nucleolus found in the nucleus [87,88,89,90]. The propensity of IDPs to aggregate is well-known (in some classic cases contributing to pathological filament structures characteristic of Parkinson’s disease or Alzheimer’s disease), but it remains to be determined how these properties of the IDPs can be leveraged to phase separate and generate these membrane-less organelle structures [90,91,92,93,94].

Our discussion here has focused on the role of IDPs in cellular signaling pathways and the unique capabilities they provide for interacting with multiple partners to coordinate signal transduction. Combining results obtained by a variety of methods provides a more complete picture of molecular function, and cross validating smFRET with other methods including NMR, EPR-DEER (electron parametric resonance—double electron-electron resonance), PRE (paramagnetic relaxation enhancement), and SAXS will provide deeper insights into IDP phenomena. Because of the different concentration ranges of these methods and the variety of modeling required in interpreting the experimental results, it is increasingly clear that molecular dynamics (MD) simulation will be essential for a complete understanding of IDP behavior and function in the future. IDPs present unique challenges for MD simulation because they are extremely sensitive to details of the force field used and they also require extensive sampling to characterize the ensemble [95,96]. Recent results suggest that methods are rapidly developing to allow MD simulation to provide insights into IDP conformational dynamics, ensemble populations and function [41,46,52,57,86,97,98,99]. Single molecule and ensemble FRET measurements combined with other methods including NMR and EPR techniques will provide valuable benchmarks to both test and constrain such simulations [41,100,101,102,103].

Understanding the mechanistic details that impact the malleability of IDPs and fine tune the binding of these ensembles to target molecules (some even remaining disordered when in the liganded state [104]) will be required before systematic approaches to modulating these conformations for engineered outcomes could be practical. Nevertheless, there already are some hints of success in therapeutic targeting of IDPs in signaling pathways by small molecules to interact with the disordered regions of proteins [2,105,106]. In a recent example, a small molecule was found to modify the interaction between DNA and the transcription factor TFIID in a way that prevented transcription initiation by RNA polymerase [2,106]. Successful detection of smFRET from IDPs inside live mammalian cells [107,108] suggests that smFRET may soon be able to reveal details of IDP coordination of signaling pathways and the mechanistic impact of such therapeutic interventions in the native cellular context. As our understanding of the molecular mechanisms that enable IDPs to coordinate major cell signaling pathways progresses, the development of additional interventions to attempt to modulate these pathways will surely follow.
